# Characterization of Two *Bovine viral diarrhea virus* Strains Originating from Cattle in Tibet, China

**DOI:** 10.1128/genomeA.01087-17

**Published:** 2017-10-19

**Authors:** Runbo Luo, Xiaoqin Wei, Hongbo Zhou, Suolang Sizhu

**Affiliations:** a Department of Animal Science, XiZang Agriculture and Animal Husbandry College, Linzhi, People’s Republic of China; b State Key Laboratory of Agriculture Microbiology, College of Veterinary Medicine, Huazhong Agricultural University, Wuhan, People’s Republic of China

## Abstract

Here, we report two strains of *Bovine viral diarrhea virus* (BVDV), named XZ01 and XZ02, that were isolated from cattle in Tibet, China. They belong to subgenotype 1b. This report will help in understanding the molecular characteristics of BVDV in Tibetan cattle.

## GENOME ANNOUNCEMENT

*Bovine viral diarrhea virus* (BVDV) is a major pathogen that may be one of the main reasons for economic losses in the livestock industry, and it affects mainly cattle (1). BVDV belongs to the genus *Pestivirus* within the family *Flaviviridae*. Based on antigenic and nucleotide differences, three species of BVDV have been recognized, BVDV-1, BVDV-2, and BVDV-3 (2, 3). In addition, based on their ability to cause a cytopathic effect (CPE), BVDV strains are further categorized as cytopathogenic (CP) or noncytopathogenic (NCP) biotypes. BVDV-1 is the dominant genotype worldwide and can be divided into at least 21 subgenotypes (BVDV-1a to BVDV-1u) (4–7).

To investigate the prevalence of BVDV in the Tibet Autonomous Region of China, a total of 920 blood serum samples were collected from different farms in Tibet, China, during 2012 to 2016 and initially screened for antibodies (Abs) to BVDV using the BVDV enzyme-linked immunosorbent assay (ELISA) kit (Feiya Biotechnology, Inc., Jiangsu, China). Then, 448 of these samples were found to be positive for BVDV infection. Positive samples were inoculated in Madin-Darby bovine kidney (MDBK) cells for 3 days. Supernatants were harvested, and viral RNA was extracted using the TRIzol RNA kit (Invitrogen, Carlsbad, CA, USA), followed by reverse transcriptase PCR (RT-PCR) with random hexanucleotide primers (Sangon Biotech, Shanghai, China). The PCR products were purified and sequenced (Sangon Biotech). Two BVDV strains (XZ01 and XZ02) were successfully isolated from these samples. The full-length coding sequence of XZ01 was 11,742 nucleotides (nt) and encoded 3,914 amino acids (aa). XZ02 contained 11,697 nt and encoded 3,899 aa. The two isolates were propagated in MDBK cells, and significant CPE was observed.

Phylogenetic analysis was performed using Molecular Evolutionary Genetics Analysis (MEGA) software, version 6.06. The full-length coding sequences of XZ01 and XZ02 were aligned with those of reference strains obtained from GenBank, including those of BVDV-1, BVDV-2, BVDV-3, and classical swine fever virus (CSFV). The bootstrap values were obtained for 1,000 replicates using the neighbor-joining (NJ) algorithm, and evolutionary distances were determined using the maximum likelihood method. The phylogenetic tree revealed that isolates XZ01 and XZ02 were classified as BVDV-1. The two genome sequences shared nucleotide sequence identities of 79% to 99% with the other BVDV-1 strains. Furthermore, the two isolates clustered with strain GX4 (GenBank accession number KJ689448), which belongs to the BVDV-1b subgenotype. To confirm the subtype assignment, a phylogenetic tree based on the N^pro^ region was constructed. For this analysis, sequences from representations of 20 BVDV-1 subtypes (i.e., subtypes -1a, -1b, -1c, -1d, -1e, -1f, -1g, -1h, -1i, -1j, -1k, -1l, -1m, -1n, -1o, -1p, -1q, -1s, -1t, and -1u) from different regions of the world were used. XZ01 and XZ02 were closely related to BVDV-1b strains GX4 and Av69 VEDEVAC (accession numbers KJ689448 and KC695814, respectively) reported in China (8) and showed about 87% to 100% sequence identity with the BVDV-1b subtype at the nucleotide level. This report will help understand the molecular characteristics of BVDV in Tibetan cattle.

### Accession number(s).

The complete coding sequences of the BVDV XZ01 and XZ02 strains were deposited in GenBank under the accession numbers MF278651 and MF278652, respectively.

## References

[1] Houe H 1999 Epidemiological features and economical importance of bovine virus diarrhoea virus (BVDV) infections. Vet Microbiol 64:89–107. doi:10.1016/S0378-1135(98)00262-4. 10028165

[2] Liu L, Xia H, Wahlberg N, Belák S, Baule C 2009 Phylogeny, classification and evolutionary insights into pestiviruses. Virology 385:351–357. doi:10.1016/j.virol.2008.12.004. 19167739

[3] Becher P, Orlich M, Shannon AD, Horner G, König M, Thiel HJ 1997 Phylogenetic analysis of pestiviruses from domestic and wild ruminants. J Gen Virol 78:1357–1366. doi:10.1099/0022-1317-78-6-1357. 9191930

[4] Deng M, Ji S, Fei W, Raza S, He C, Chen Y, Chen H, Guo A 2015 Prevalence study and genetic typing of bovine viral diarrhea virus (BVDV) in four bovine species in China. PLoS One 10:e0121718. doi:10.1371/journal.pone.0121718. 25849315 PMC4388703

[5] Yeşilbağ K, Förster C, Ozyiğit MO, Alpay G, Tuncer P, Thiel HJ, König M 2014 Characterisation of bovine viral diarrhoea virus (BVDV) isolates from an outbreak with haemorrhagic enteritis and severe pneumonia. Vet Microbiol 169:42–49. doi:10.1016/j.vetmic.2013.12.005. 24447942

[6] Giammarioli M, Ceglie L, Rossi E, Bazzucchi M, Casciari C, Petrini S, De Mia GM 2015 Increased genetic diversity of BVDV-1: recent findings and implications thereof. Virus Genes 50:147–151. doi:10.1007/s11262-014-1132-2. 25349062

[7] Xue F, Zhu YM, Li J, Zhu LC, Ren XG, Feng JK, Shi HF, Gao YR 2010 Genotyping of bovine viral diarrhea viruses from cattle in China between 2005 and 2008. Vet Microbiol 143:379–383. doi:10.1016/j.vetmic.2009.11.010. 20036078

[8] Xie Z, Fan Q, Xie Z, Liu J, Pang Y, Deng X, Xie L, Luo S, Khan MI 2014 Complete genome sequence of a bovine viral diarrhea virus strain isolated in southern China. Genome Announc 2(3):e00512-14. doi:10.1128/genomeA.00512-14. 24948756 PMC4064797

